# Laparoscopic splenectomy for solitary splenic metastasis in a patient with ovarian cancer with a long disease-free interval: a case report

**DOI:** 10.1186/s13256-018-1673-4

**Published:** 2018-05-15

**Authors:** Katsuhiko Yasuda, Tomoo Yoshimura, Hiroaki Kitade, Hidesuke Yanagida, Naoki Hosaka

**Affiliations:** 1 Department of Obstetrics and Gynecology, Kansai Medical University Medical Center, 10-15 Fumizono-cho, Moriguchi, Osaka 570-8507 Japan; 2 Department of Surgery, Kansai Medical University Medical Center, Moriguchi, Osaka Japan; 3 Department of Pathology, Fuchu Hospital, Izumi, Osaka Japan

**Keywords:** Laparoscopy, Metastasis, Ovarian cancer, Spleen

## Abstract

**Background:**

In general, splenic metastasis of epithelial ovarian cancer is considered a terminal stage resulting in widespread metastasis. Solitary splenic metastasis of epithelial ovarian cancer is rare in patients with post-treatment ovarian cancer with long disease-free intervals.

**Case presentation:**

We report a case of a 62-year-old Japanese woman who presented with elevated serum cancer antigen 125 due to a solitary splenic metastasis of ovarian cancer. She underwent primary open cytoreduction including resection of the right ovarian cancer and postoperative chemotherapy, followed by secondary open cytoreduction and additional postoperative chemotherapy. The disease-free interval was more than 5 years after the additional postoperative chemotherapy. She did not complain of any symptoms and there were no abnormal findings except for elevated cancer antigen 125. However, computed tomography and magnetic resonance imaging revealed a tumor of 6.5 × 4.5 cm in her spleen, and ^18^F-fluorodeoxyglucose positron emission tomography-computed tomography showed no other metastatic lesions. Laparoscopic splenectomy was performed as tertiary cytoreduction with a diagnosis of a solitary splenic metastasis. Her elevated cancer antigen 125 immediately decreased to within the normal range after the splenectomy. On microscopic examination, the tumor was grade 3 endometrioid adenocarcinoma localized in the spleen, consistent with the previous grade 3 endometrioid adenocarcinoma ovarian cancer.

**Conclusions:**

Elevated cancer antigen 125 is useful for early detection of metastasis of ovarian cancer. Computed tomography, magnetic resonance imaging, and ^18^F-fluorodeoxyglucose positron emission tomography-computed tomography are useful to evaluate whether splenic metastasis of ovarian cancer is solitary, and laparoscopic splenectomy is safe and feasible for a solitary splenic metastasis.

## Background

According to the literature, metastasis in the spleen occurs in approximately 1% of malignant tumors [1], and colorectal and ovarian cancer are known to metastasize to the spleen more frequently than other malignant tumors [2]. Epithelial ovarian cancer is known to metastasize throughout the peritoneum to cause visceral spreading, including to the capsule of the spleen. Therefore, splenic metastasis of epithelial ovarian cancer is usually found in patients at the terminal stage. A solitary splenic metastasis of epithelial ovarian cancer is very rare and considered to have metastasized hematogenously. Although the role of cytoreduction in the metastasis of ovarian cancer is not well established, cytoreduction can improve the oncological outcome in platinum-sensitive patients with solitary metastasis to the tissues or organs, including the spleen [3, 4].

We present a rare case of a solitary splenic metastasis of epithelial ovarian cancer, which occurred after a 5-year disease-free interval, and laparoscopic splenectomy as tertiary cytoreduction for the metastatic tumor.

## Case presentation

A 62-year-old Japanese woman was referred to our hospital because of a pelvic tumor with massive ascites, which was diagnosed as an ovarian tumor by pelvic examination, vaginal ultrasonography, and magnetic resonance imaging (MRI). Her blood cancer antigen 125 (CA125) level was as high as 578.6 U/mL (reference range, 0–35 U/mL). First, she underwent probe laparotomy, including resection of the right ovarian tumor and partial resection of her omentum in July 2010 because of the massive adhesion similar to a frozen pelvis. Pathological examination revealed grade 3 endometrioid adenocarcinoma. A tumor > 2 cm with the same pathological diagnosis was confirmed in the resected omentum. She was diagnosed as having abdominal and pelvic cavity metastases of ovarian cancer, International Federation of Gynecology and Obstetrics (FIGO) stage IIIC, and received six cycles of systemic chemotherapy: paclitaxel and carboplatin (TC) scheme. She received 175 mg/m^2^ TC, according to an area under the curve (AUC) of 5, for 21 days in each cycle. During the chemotherapy, her CA125 level decreased from 578.6 U/mL to 10.7 U/mL. Follow-up MRI demonstrated that the solid tumor and ascites had disappeared completely. Thus, the post-chemotherapy evaluation was complete remission.

Subsequently, she underwent total abdominal hysterectomy, left salpingo-oophorectomy, and resection of the residual omentum in February 2011. The pathology of the left ovarian tumor was also grade 3 endometrioid adenocarcinoma. However, no residual adenocarcinoma was found in her pelvic cavity or her omentum. Therefore, the second cytoreduction was considered the optimal surgery. Another six cycles of systemic TC chemotherapy were administered after the second operation. Our patient received the last cycle of chemotherapy in July 2011.

**Fig. 1:**
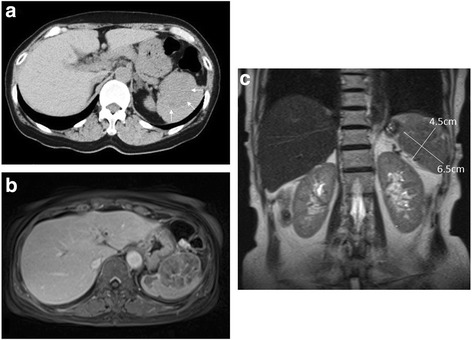
Computed tomography and magnetic resonance imaging examination. **a** Computed tomography performed in December 2016 shows a pale homogenous tumor in the spleen. **b** Enhanced magnetic resonance imaging performed in January 2017 shows a tumor with internal heterogeneity and irregular margin in the spleen. The capsule of the spleen was intact. **c** Coronal magnetic resonance imaging view shows a 6.5 × 4.5 cm tumor localized in the spleen. *White arrows* indicate a tumor in the spleen

She did well with no complaints and had a normal CA125 level until November 2016, when she was found to have an elevated CA125 level of 65.4 U/mL. However, a pelvic examination with vaginal ultrasonography did not show any abnormal findings. After 1 month, her CA125 level was still high, at 67.8 U/mL. In November 2016, her liver enzymes, such as glutamic oxaloacetic transaminase (GOT), glutamic pyruvic transaminase (GPT), and gamma-glutamyltransferase (GGT), were within normal range, and each value was 22, 12, and 12 IU/L, respectively. A month later, GOT, GPT, and GGT were still within normal range. Computed tomography (CT) of her abdomen and pelvic cavity revealed a pale hypodense area in the spleen (Fig. 1a).

**Fig. 2:**
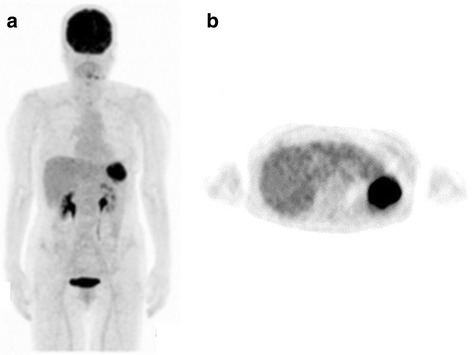
^18^F-fluorodeoxyglucose positron emission tomography-computed tomography examination. **a** ^18^F-fluorodeoxyglucose positron emission tomography-computed tomography performed in February 2017 shows neither abnormal hypermetabolic lesions nor lymph node swelling in the whole body except for the left upper abdominal lesion. **b** Axial ^18^F-fluorodeoxyglucose positron emission tomography-computed tomography view shows a solitary hypermetabolic lesion in the spleen

MRI revealed a 6.5 × 4.5 cm tumor with irregular margins in her spleen, which suggested splenic metastasis of ovarian cancer (Fig. 1b, c). ^18^F-fluorodeoxyglucose positron emission tomography (FDG-PET)-CT performed in February 2017 revealed significant accumulation of radiolabeled glucose only in her spleen (Fig. 2a, b). Therefore, we diagnosed the splenic tumor as an isolated splenic metastasis of ovarian cancer.

Our patient underwent laparoscopic splenectomy in March 2017. Under a right lateral position, 12 mm ports were inserted at her navel after minimal laparotomy. Three ports (5 mm in diameter) were added from the left upper quadrant area. The tumor did not invade outside the spleen, and no peritoneal dissemination was observed. We divided the splenocolic ligament, the lienorenal ligament, and the splenogastric ligament, and mobilized her spleen. The hilum of her spleen was exposed and the tail of her pancreas was identified. Her splenic artery and vein were dissected and divided using an Echelon stapler. The spleen was extracted from the extended 12-mm port site.

**Fig. 3:**
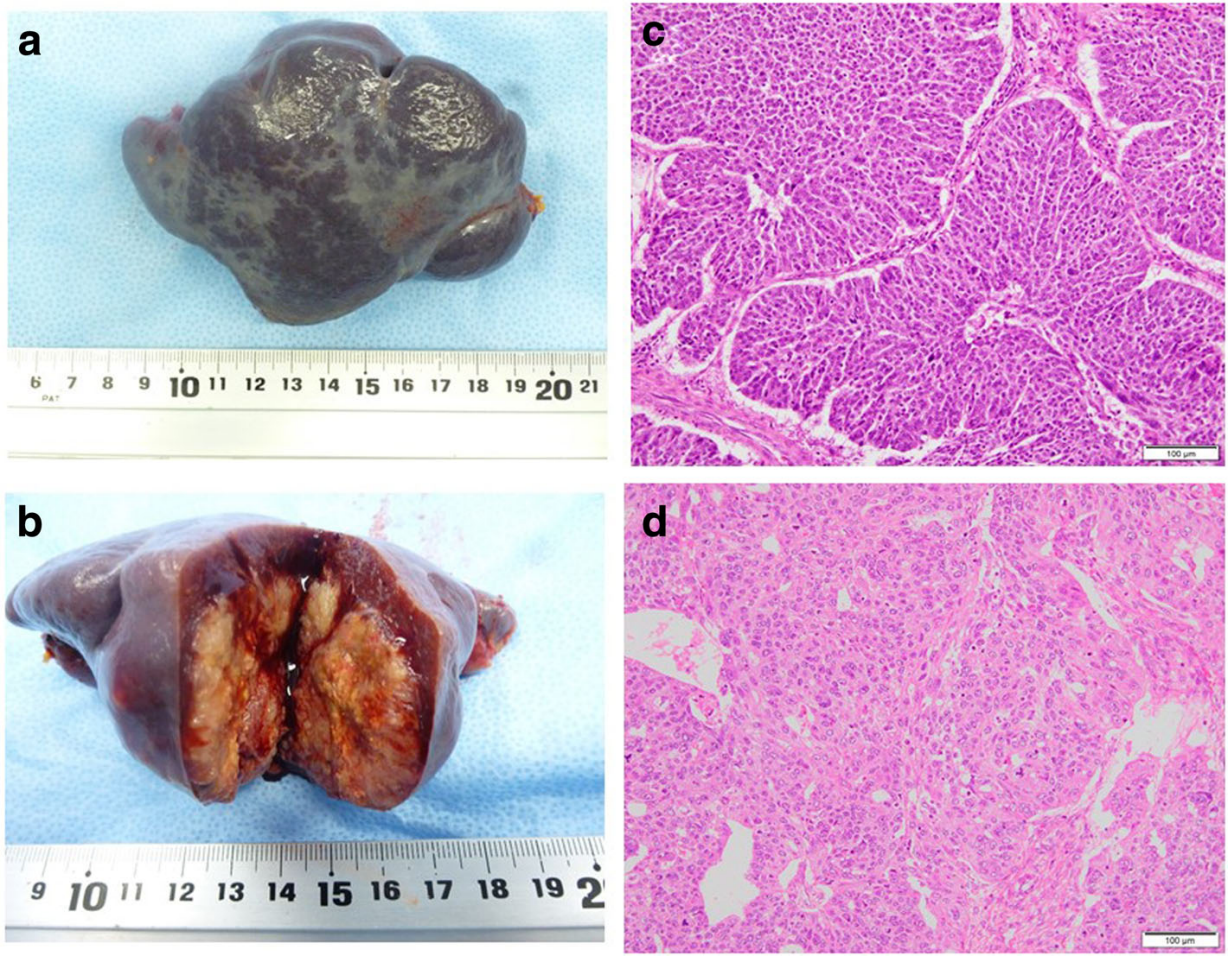
Macroscopic findings and histopathological examination. **a** There was no macroscopic invasion of the splenic capsule. **b** The gross specimen included a single, oval, yellow-white, solid tumor. **c** Histopathological examination of the splenic tumor at laparoscopic tertiary cytoreduction in 2017 indicated grade 3 endometrioid adenocarcinoma with similar features to the primary ovarian tumor (hematoxylin and eosin, × 200 original magnification). **d** Microscopic features of the ovarian tumor at primary cytoreduction in 2010 indicated grade 3 endometrioid adenocarcinoma (hematoxylin and eosin, × 200 original magnification)

Pathological examination revealed grade 3 endometrioid adenocarcinoma localized in the spleen, consistent with the previous grade 3 endometrioid adenocarcinoma ovarian cancer (Fig. 3a, b). Her postoperative CA125 level decreased to 18.1 U/mL. Her postoperative course was uneventful, and her liver function and kidney function were within normal range 1 month after laparoscopic splenectomy. She received 175 mg/m^2^ paclitaxel, AUC 5 carboplatin, and 15 mg/kg bevacizumab as postoperative chemotherapy. Carboplatin was omitted in the third to sixth courses of chemotherapy because of an allergy to carboplatin in the second course. After the sixth course of chemotherapy she underwent a clinical examination, CA125 assessment, abdominal ultrasonography, and abdominal CT. All tests showed no recurrence of ovarian cancer.

## Discussion

We experienced a solitary splenic metastasis of epithelial ovarian cancer occurring after a 5-year disease-free interval and successful laparoscopic splenectomy. Generally, splenic metastasis is known to occur from hematological malignant diseases such as malignant lymphoma and leukemia. However, splenic metastasis is sometimes found in patients with end stage cancer as multiorgan metastasis. Colorectal and ovarian cancer are sometimes known to cause splenic metastasis [2]. Splenic metastasis of ovarian cancer is generally associated with peritoneal spreading with multiorgan involvement. Therefore, solitary splenic metastasis is rare, and metastasis is considered to occur through the hematogenic route. Recently, 35 cases of splenic metastasis of ovarian cancer were reviewed in the literature, 30 of which were solitary metastases [5]. On pathological examination, 28 cases were serous adenocarcinoma, 1 case was angiosarcoma, and 1 case was carcinosarcoma. According to some studies, including the previously mentioned study, the time to development of postoperative splenic metastasis varies, and eight cases were found after more than 5 years [6–12]. The longest time to development observed thus far is 20 years [7]. On pathological examination, seven cases were serous adenocarcinoma and one case was grade 3 endometrioid adenocarcinoma. Our case was also a solitary splenic metastasis of ovarian cancer with grade 3 endometrioid adenocarcinoma that occurred after a 5-year disease-free interval. Therefore, our results suggest that grade 3 endometrioid adenocarcinoma ovarian cancer is likely to cause solitary splenic metastasis after more than 5 years, even though previous studies have found that the majority of solitary splenic metastases of ovarian cancer with a disease-free interval of longer than 5 years were caused by serous adenocarcinoma. A solitary splenic metastasis of ovarian cancer is treated by open or laparoscopic splenectomy followed by chemotherapy, and the prognosis is generally good. According to PubMed, the first reported laparoscopic splenectomy for solitary splenic metastasis was a hand-assisted laparoscopic splenectomy in 1998 [13]. After that, the number of laparoscopic splenectomies has increased, and they have been reported to be safe and feasible [14]. Although our case involved laparoscopic splenectomy as tertiary cytoreduction, the operation was performed successfully and the postoperative course was uneventful. Recently, another case of laparoscopic splenectomy as quaternary cytoreduction was reported to have been performed successfully [15].

The majority of solitary splenic metastases of ovarian cancer are asymptomatic. Therefore, it is difficult to diagnose solitary splenic metastasis early. However, serum CA125 is useful to diagnose asymptomatic solitary splenic metastasis of ovarian cancer because the majority of cases show an increase in serum CA125 level [15]. Our case also showed no symptoms, but the serum CA125 level was elevated before imaging, indicating the presence of the splenic metastasis. In addition, the elevated serum CA125 level decreased to a normal level immediately after the operation. CT or MRI is essential to diagnose solitary splenic metastasis of ovarian cancer, and FDG-PET-CT may add more particular findings about the metastasis of ovarian cancer. We diagnosed the solitary splenic metastasis of ovarian cancer by using CT and MRI, and confirmed our diagnosis with FDG-PET-CT. Therefore, we were able to opt for laparoscopic splenectomy rather than open splenectomy. Finally, we reconfirmed the solitary metastatic tumor of the ovarian cancer pathologically.

## Conclusions

We believe that solitary splenic metastasis occurs from ovarian cancer even when the disease-free interval is more than 5 years, and that not only serous adenocarcinoma but also endometrioid adenocarcinoma can cause solitary splenic metastasis. Serum CA125 level may be useful for diagnosing asymptomatic solitary splenic metastasis, and diagnostic imaging tools, especially FDG-PET-CT, may be useful in evaluating whether the splenic metastasis is solitary. Laparoscopic splenectomy as multiple cytoreduction may be safe and feasible for solitary splenic metastasis of ovarian cancer.

## Abbreviations

AUC: Area under the curve
CA125: Cancer antigen 125
CT: Computed tomography
FDG-PET: ^18^F-fluorodeoxyglucose positron emission tomography
GGT: Gamma-glutamyltransferase
GOT: Glutamic oxaloacetic transaminase
GPT: Glutamic pyruvic transaminase
MRI: Magnetic resonance imaging
TC: Paclitaxel and carboplatin
